# The Population Problem

**Published:** 1915-12-11

**Authors:** Binnie Dunlop

**Affiliations:** 24 Alexandra Court, S.W.


### THE EDITOR'S LETTER-BOX.

### "The Population Problem."

*To the Editor of* The Hospital.

Sir,—It is true that it is "officially on record," as you well say, that if the birth-rate had remained as high as it was in 1876 we should have had 467,837 more infants born in 1914. But might we not also have had at least that number more of deaths? In other words, would the death-rate have fallen if the birth-rate had not fallen? The decline of the death-rate is "officially" attributed mainly to progress in sanitation and medical treatment. But if it is rightly so attributed, why has the death-rate not fallen in Ireland? The truth is that we still only find a falling death-rate in those countries where there is a falling birth-rate. Infant-saving and endowment of motherhood schemes ought, therefore, to include the encouragement of parental prudence among the poor and instruction in birth-control. I shall be glad to send information on the subject to any of your readers.—Yours sincerely, Binnie Dunlop, M.B., Ch.B.

24 Alexandra Court, S.W.

